# Associations of neighborhood racial and ethnic, economical, and educational segregation with breast cancer risk: The Multiethnic Cohort Study

**DOI:** 10.1007/s10552-026-02187-4

**Published:** 2026-07-24

**Authors:** Ugonna Ihenacho, Jenna Khan-Gates, Katherine Lin, Janine V. Abe, Pushkar P. Inamdar, Juan Yang, Yuqing Li, Meera Sangaramoorthy, Christopher A. Haiman, Loïc Le Marchand, Lynne R. Wilkens, Scarlett Lin Gomez, Salma Shariff-Marco, Anna H. Wu, Iona Cheng

**Affiliations:** 1 Department of Population and Public Health Sciences, Keck School of Medicine, University of Southern California, 1450 Biggy Street, Los Angeles, CA 90032 USA; 2 Population Sciences in the Pacific Program, University of Hawai‘i Cancer Center, 701 Ilalo St, Honolulu, HI 96813 USA; 3 Department of Epidemiology and Biostatistics, School of Medicine, University of California San Francisco, 550 16th Street, Second Floor, San Francisco, CA 94158 USA; 4 Helen Diller Family Comprehensive Cancer Center, University of California San Francisco, 550 16th Street, Second Floor, San Francisco, CA 94158 USA

**Keywords:** Female, Breast cancer, Incidence, Neighborhood, Residential segregation, Health disparities, Cohort studies

## Abstract

**Purpose:**

Residential segregation is recognized as an upstream driver of poor health, yet few studies have evaluated its impact on breast cancer incidence at a local level. We examined associations of neighborhood residential segregation with breast cancer (BC) incidence in the Multiethnic Cohort Study (MEC).

**Methods:**

Measures of Index of Concentration at the Extremes were developed to assess residential segregation by income, education, race and ethnicity, and racialized income. A prospective study (1993–1996 through December 2019) was conducted to examine associations of these measures and breast cancer incidence for 101,785 African American, Japanese American, Latina, Native Hawaiian, and White female participants in the MEC, aged 45–75 years at baseline and residing in California and Hawai‘i. Multivariable Cox regression was conducted to evaluate the associations of several residential segregation measures with breast cancer incidence (cases = 7,381) adjusting for demographic, lifestyle, and reproductive factors.

**Results:**

In California, BC risk was higher for females residing in neighborhoods with the highest compared to lowest concentration of privilege by income segregation (quintile 5 [Q5] vs. Q1: hazard ratio [HR] 1.12, 95% confidence interval [CI] 1.00–1.25, *P*_trend_ = 0.001) and education segregation (Q5 vs. Q1: HR 1.15, 95% CI 1.01–1.31; *P*_trend_ = 0.01). For Latina females, risks increased with increasing privilege for measures of income, education, race and ethnicity, and racialized income residential segregation (*P*_trends_ < 0.05). No associations were observed in Hawai‘i.

**Conclusion:**

In California, residential economic privilege was independently associated with increased BC risk. Future studies should examine neighborhood- and individual-level pathways placing females residing in privileged areas at greater risk.

**Supplementary Information:**

The online version contains supplementary material available at https://doi.org/10.1007/s10552-026-02187-4.

## Introduction

Breast cancer is the most common cancer diagnosed among females in the United States (US) with increasing incidence rates that vary across racial and ethnic groups [1]. In 2017–2021, age adjusted breast cancer incidence rates (AAIRs) were highest among non-Latina White (AAIR 137.9 per 100,000 females) and African American females (AAIR 131.3) but there is a growing burden among other racial and ethnic groups [1]. In 2012–2021, the increase in breast cancer incidence among Latina females (1.6% per year) was about twice that of White and African American females (1.0% and 0.8% per year, respectively) but the highest increase was observed among Asian American, Native Hawaiian and Pacific Islander females (2.6% per year) [1]. When disaggregating Asian American, Native Hawaiian and Pacific Islander populations into distinct ethnic groups, prior studies have demonstrated Native Hawaiian females have the highest breast cancer incidence across all racial and ethnic groups, approximately 10–50% higher than non-Latina White females [2–4].

There is increasing evidence that cancer health inequities are influenced by the neighborhood context [5–7], which is shaped by structural and social drivers of health. Residential segregation is the geographic separation of different subgroups within a population by sociodemographic factors such as race, ethnicity, and income. It has been associated with poor breast cancer outcomes that can be driven by neighborhood disinvestment and limited access to healthcare, including an increased risk of estrogen receptor negative breast cancer, late stage at diagnosis, more aggressive tumor characteristics, and increased mortality [7–14]. Few studies have examined residential racial, ethnic, and economic segregation as a contributor to breast cancer incidence [15–17]. Segregation may contribute to breast cancer risk through its effects on behavioral risk factors such as reduced physical activity, and increased obesity, smoking and alcohol use [18], as well as chronic stress and its effect on the neuroendocrine system and immune function [19, 20].

Most studies of residential segregation and cancer employ segregation measures at city-level or at larger geographic areas with race and ethnicity and economic segregation created as separate measures. The Index of Concentration at the Extremes, however, can be applied to smaller geographic levels (e.g., census tracts) and used to consider the joint residential segregation by race, ethnicity and income with the measure of racialized income (i.e., racialized economic segregation). The Index of Concentration at the Extremes is a measure of spatial social polarization that was first developed in 2001 [21] to quantify the proportion of a population concentrated at the extremes of privilege and deprivation in neighborhoods. It has been used extensively by Krieger and colleagues to study associations of socioeconomic (education, income), racial and ethnic, and racialized economic segregation with black carbon exposure, violent crime, COVID-19, cancer incidence, breast cancer estrogen receptor status, and cause-specific mortality [15, 16, 22–26]. Of the studies that have examined the association between the Index of Concentration at the Extremes measures and breast cancer incidence [15–17], only one was population-based using Massachusetts cancer registry data; results showed lower incidence rates among female residents living in neighborhoods with greater concentrations of low-income households compared to high-income households (income segregation quintile 1 [Q1] vs. Q5: incidence rate ratio [IRR] 0.86; 95% confidence interval [CI] 0.82–0.91) and among those in neighborhoods with greater concentrations of low-income African American households versus high-income White households (racialized income segregation Q1 vs. Q5: IRR 0.89; 95% CI 0.84–0.94) [15]. Although this Massachusetts study demonstrated the utility of evaluating breast cancer incidence with the measures of Index of Concentration at the Extremes, study subjects were mostly White females (76%) and established lifestyle or reproductive breast cancer risk factors were not accounted for [15].

In this prospective study, we examined the associations of residential segregation as measured by the Index of Concentration at the Extremes and breast cancer incidence among 101,785 female Multiethnic Cohort Study (MEC) participants from California and Hawai‘i. Specifically, we developed small area census tract measures of economic segregation, racial and ethnic segregation, and racialized economic segregation following the approach of prior studies [15, 16, 22, 23, 26, 27]. Our study is the first to develop residential segregation measures using the Index of Concentration at the Extremes for Native Hawaiian populations, therefore expanding upon current metrics and offering insights for the role of local segregation in breast cancer risk across five racial and ethnic populations.

## Materials and methods

### Study subjects

The MEC is a large, population-based prospective cohort study designed to investigate diet and cancer with over 215,000 participants from California (primarily Los Angeles County) and Hawai‘i [28]. The cohort mainly consists of self-reported African American, Japanese American, Native Hawaiian, Latino, and White participants aged 45–75 years at enrollment in 1993–1996 [28]. Driver’s license records in California and Hawai‘i were sampled to ensure socioeconomic diversity in each racial and ethnic group. Additionally, voter registration files were used in Hawai‘i to supplement identification for older Japanese American women. In Los Angeles County, we identified census tracts with ≥ 65% African American residents, obtained a list of African American residents in California ages ≥ 65 years from Health Care Financing Administration (HCFA) files, and used driver’s license records to selectively sample and recruit African American study participants. Ethnic-specific surname lists and Japanese first-name lists were used to recruit Latino participants and female Japanese American participants. Self-administered paper questionnaires were mailed up to three times to solicit participation. Completion of a comprehensive 26-page mailed baseline questionnaire regarding demographic, anthropometric, lifestyle, reproductive, family history, and other risk factors indicated consent and enrollment into the study.

Incident invasive breast cancer cases were identified by annual linkages with California and Hawai‘i Surveillance, Epidemiology, and End Results Program (SEER) cancer registries (n = 7,381 breast cancer cases). Of the 118,748 eligible female MEC participants who completed a baseline survey, we excluded those who did not self-identify with one of these five racial and ethnic groups (n = 7,725), had breast cancer prior to cohort entry according to self-report or cancer registry linkage (n = 5,268), did not have a geocodeable address (n = 3,964), or had an invalid follow-up time (n = 6). The final study population included 56,856 female participants from California and 44,929 from Hawai‘i. Follow-up time started from date of cohort entry (1993–1996) to the earliest date of invasive breast cancer diagnosis, death, or study end date (12/31/2019) with a median (interquartile range) follow-up of 24.5 (15.8–25.8) years. Institutional Review Boards at the University of Hawai‘i Cancer Center, University of Southern California, and University of California, San Francisco approved the study protocol.

### Index of Concentration at the Extremes

We developed residential segregation measures of the Index of Concentration at the Extremes to assess the extent of an area’s population that is characterized with the highest concentration of privilege versus lowest concentration of privilege, calculated as [21]:

$$ICEi\, = \,\left( {Ai{-}Pi} \right)/Ti$$

Where *A*_*i*_ = the number of people or households classified as most privileged in area *i*, *P*_*i*_ = the number of people or households classified as least privileged in area *i*, and *T*_*i*_ = the total population or households in area *i* with household data. Values for the residential segregation measures of the Index of Concentration at the Extremes (*ICE*_*i*_) ranged from −1 to + 1 representing all people or households in the least privileged group to all people or households in the most privileged group, respectively, and a value of 0 representing balance of the extremes or no people or households in the extremes. The estimate of privilege can be modeled as ranked categories (i.e., quantiles) [24].

Baseline residential addresses (1993–1996) were geocoded to geographic coordinates using parcel and street centerline data and linked to 1990 US Census tracts for each state as the neighborhood unit. Separate residential segregation measures were created for household income, education, race and ethnicity, and household income by race and ethnicity (racialized income) with 1990 US Census tract-level data for Hawai‘i and California to allow for the assessment of the residential segregation measures with a localized, small-area measure. Table 1 provides the criteria and terminology based on the 1990 US Census for defining the most and least privileged group (*A*_*i*_ and *P*_*i*_, respectively) within each census tract for the residential segregation measures evaluated. The residential segregation measures were categorized into quintiles based on the regional distributions for Los Angeles County or Hawai‘i; Quintile 1 represents the least privileged and Quintile 5 represents the most privileged group in the population. The racialized income and race and ethnicity segregation measures are constructed for minoritized race-specific populations (i.e., Non-Hispanic Black, Non-Hispanic Asian American or Pacific Islander, or Hispanic households) using Non-Hispanic White households as the comparison group. Therefore, there are no race-specific segregation measures for the Non-Hispanic White population.

**Table 1:** Residential Segregation as measured by the Index of Concentration at the Extremes and their components

Measure	Most and least privileged criteria (*A*_*i*_ and *P*_i_)	Denominator (*T*_*i*_)
Income segregation^a^	Number of households classified as most (*A*_*i*_) or least (*P*_*i*_) privileged where the household income was within the highest or lowest quintile, respectively, of the household income distribution of the state of CA or HI	Total number of households with income data
Education segregation	Persons aged 25 or older with 4 or more years of college education were classified as most privileged (*Ai*), those with less than a high school education as the least privileged (*P*_*i*_)	All persons aged 25 or older
Racialized income segregation^a,b,c^	White households with income in the top quintile of the state (CA or HI) were classified as the most privileged (*A*_*i*_) and Black, Asian American or Pacific Islander^d^, or Hispanic households in the lowest household income quintile for the state (CA or HI) as the least privileged (*P*_*i*_)	Total number of households with income and race data
Race and ethnicity segregation^b,e^	Non-Hispanic White persons were classified as the most privileged (*A*_*i*_) and Non-Hispanic Black, Non-Hispanic Asian American or Pacific Islander^d^, Hispanic, Japanese American, or Native Hawaiian persons classified as the least privileged (*P*_*i*_), separately for each measure	Total population with race and ethnicity data

Residential segregation measures are calculated by census tract (area unit *i*, based on 1990 US Census data) as: *ICE*_*i*_ = *(A*_*i*_*–P*_*i*_*)/T*_*i*_

Abbreviations: *A*_*i*_, the number of people or households classified as most privileged in area *i*; CA, California; HI, Hawai‘i; *ICE*_*i*_, calculated Index of Concentration at the Extremes residential segregation measure in area *i*; *P*_*i*_, the number of people or households classified as least privileged in area *i*; *T*_*i*_, Total number of households in area *i* with data.

^a^ The income cutoffs for CA and HI were as follows: highest quintile, income ≥ $75,000; lowest quintile, income < $15,000.

^b^ When referring to study participants, we use the terms “Latino” and “African American,” as per the study’s terminology. However, we utilize the 1990 US Census terminology, “Hispanic” and “Black” to reflect the source of the data when discussing the residential segregation measures.

^c^ Ethnicity was not reported in conjunction with race data for household income data in the 1990 US Census, therefore race groups are reported without regard to Hispanic ethnicity and Hispanic ethnicity is reported without regard to race.

^d^ “Asian American or Pacific Islander” populations are evaluated as one combined racial and ethnic group.

^e^ For HI measures, Japanese American and Native Hawaiian groups are reported without regard to Hispanic ethnicity.

### Statistical analysis

Invasive breast cancer incidence was modeled using Cox proportional hazards regression with age as the time scale, accounting for clustering at the census tract-level [29]. Hazard ratios (HRs) and 95% CIs were estimated for minimally adjusted models with age at cohort entry in five-year categories and race and ethnicity as strata variables. Fully adjusted models further included education, first degree family history of breast cancer, age at menarche, age at birth of first child, number of children, menopausal status, menopausal hormone use, alcohol use, smoking status, daily energy intake, physical activity, body mass index, and weight change from age 21 to baseline age. The proportional hazards assumption was assessed using cross-product interaction terms for each covariate with time; no serious violations were observed. Race and ethnicity-specific analyses were conducted for race and ethnicity and racialized income segregation measures. Stratified analyses were conducted by race and ethnicity and also by state to account for structural, social, political, and economic differences that may contribute to heterogeneity in the residential segregation measures [2, 30, 31]. Separate models by breast cancer hormone receptor status (hormone receptor positive: estrogen receptor [ER] or progesterone receptor [PR] positive, hormone receptor negative: ER and PR negative), and stage (localized, regional & distant) were conducted; differences by hormone receptor status and stage were assessed by a competing risk analysis using the Lunn-McNeil approach [32]. Tests for linear trend were estimated by including the ordinal residential segregation measures as a continuous measure in regression models. All hypotheses were two-sided with a significance level of *P* < 0.05. Analyses were completed using SAS software version 9.4 (SAS Institute Inc., Cary, NC).

## Results

### Study population

Overall, there were 56,856 female MEC participants residing in California at baseline, who self-identified as African American (35.7%), Japanese American (10.5%), Latina (38.3%), Native Hawaiian (0.1%), or White (15.3%) race and ethnicity (Table 2). Latina females were more likely to be a high school graduate or less compared to females of the other racial and ethnic groups. Japanese American and White females were more likely to be nulliparous, older at age at first birth, and current menopausal hormone users at baseline compared to African American and Latina females. Overall, 44,929 female MEC participants resided in Hawai‘i at baseline and self-identified as Japanese American (48.2%), Native Hawaiian (16.5%), or White (35.3%) race and ethnicity. Japanese American females were older and less likely to be alcohol consumers or ever smokers compared to Native Hawaiian and White females. Native Hawaiian females were younger, had a younger age of first birth and menarche, and had a greater increase in weight from age 21 to baseline.

**Table 2:** Distribution of breast cancer risk factors by state and race and ethnicity among Multiethnic Cohort female participants, 1993–2019

	California					Hawai‘i			
	Overall	African American	Japanese American	Latina	White	Overall	Japanese American	Native Hawaiian	White
N (%)	n = 56,856	n = 20,319	n = 5,983	n = 21,787	n = 8,695	n = 44,929	n = 21,659	n = 7,391	n = 15,879
Race and ethnicity									
African American	20,319 (35.7)					0 (0)			
Japanese American	5,983 (10.5)					21,659 (48.2)			
Latina	21,787 (38.3)					0 (0)			
Native Hawaiian	72 (0.1)					7,391 (16.5)			
White	8,695 (15.3)					15,879 (35.3)			
Age at baseline (years)									
45–49	7,524 (13.2)	3,052 (15.0)	746 (12.5)	2,710 (12.4)	1,000 (11.5)	9,925 (22.1)	3,630 (16.8)	2,198 (29.7)	4,097 (25.8)
50–54	7,985 (14.0)	2,947 (14.5)	729 (12.2)	3,233 (14.8)	1,063 (12.2)	7,535 (16.8)	2,895 (13.4)	1,481 (20.0)	3,159 (19.9)
55–59	10,692 (18.8)	3,008 (14.8)	906 (15.1)	5,094 (23.4)	1,665 (19.2)	6,029 (13.4)	2,692 (12.4)	1,135 (15.4)	2,202 (13.9)
60–64	10,633 (18.7)	2,820 (13.9)	1,056 (17.7)	5,004 (23.0)	1,736 (20.0)	6,797 (15.1)	3,713 (17.1)	1,032 (14.0)	2,052 (12.9)
65–69	9,984 (17.6)	4,045 (19.9)	1,100 (18.4)	3,240 (14.9)	1,595 (18.3)	7,408 (16.5)	4,410 (20.4)	865 (11.7)	2,133 (13.4)
70–74	8,576 (15.1)	3,757 (18.5)	1,184 (19.8)	2,215 (10.2)	1,419 (16.3)	5,963 (13.3)	3,552 (16.4)	542 (7.3)	1,869 (11.8)
75 +	1,462 (2.6)	690 (3.4)	262 (4.4)	291 (1.3)	217 (2.5)	1,272 (2.8)	767 (3.5)	138 (1.9)	367 (2.3)
Education									
≤ High school graduate	29,988 (52.7)	8,433 (41.5)	2,137 (35.7)	15,889 (72.9)	3,500 (40.3)	17,496 (38.9)	9,519 (44.0)	4,065 (55.0)	3,912 (24.6)
Some college	16,114 (28.3)	7,267 (35.8)	2,153 (36.0)	3,847 (17.7)	2,817 (32.4)	13,224 (29.4)	5,801 (26.8)	2,082 (28.2)	5,341 (33.6)
College graduate	5,106 (9.0)	2,230 (11.0)	1,019 (17.0)	757 (3.5)	1,093 (12.6)	6,799 (15.1)	3,117 (14.4)	614 (8.3)	3,068 (19.3)
Graduate and professional school	4,722 (8.3)	2,070 (10.2)	618 (10.3)	842 (3.9)	1,188 (13.7)	7,046 (15.7)	3,039 (14.0)	565 (7.6)	3,442 (21.7)
Missing	926 (1.6)	319 (1.6)	56 (0.9)	452 (2.1)	97 (1.1)	364 (0.8)	183 (0.8)	65 (0.9)	116 (0.7)
Family history of breast cancer in mother or sisters									
No	46,578 (81.9)	16,461 (81.0)	5,106 (85.3)	17,864 (82.0)	7,086 (81.5)	38,175 (85.0)	18,550 (85.7)	6,040 (81.7)	13,585 (85.6)
Yes	5,708 (10.0)	2,173 (10.7)	582 (9.7)	1,838 (8.4)	1,109 (12.8)	4,979 (11.1)	2,204 (10.2)	950 (12.9)	1,825 (11.5)
Missing	4,570 (8.0)	1,685 (8.3)	295 (4.9)	2,085 (9.6)	500 (5.8)	1,775 (4.0)	905 (4.2)	401 (5.4)	469 (3.0)
Age at menarche									
≤ 12 years	26,864 (47.3)	9,790 (48.2)	2,716 (45.4)	10,011 (46.0)	4,307 (49.5)	22,464 (50.0)	10,537 (48.7)	4,138 (56.0)	7,789 (49.1)
13–14 years	21,453 (37.7)	7,420 (36.5)	2,362 (39.5)	8,288 (38.0)	3,358 (38.6)	16,815 (37.4)	8,157 (37.7)	2,345 (31.7)	6,313 (39.8)
> 14 years	7,392 (13.0)	2,605 (12.8)	815 (13.6)	3,040 (14.0)	927 (10.7)	5,107 (11.4)	2,688 (12.4)	785 (10.6)	1,634 (10.3)
Missing	1,147 (2.0)	504 (2.5)	90 (1.5)	448 (2.1)	103 (1.2)	543 (1.2)	277 (1.3)	123 (1.7)	143 (0.9)
Age at birth of first child									
Nulliparous	6,766 (11.9)	2,691 (13.2)	1,020 (17.1)	1,762 (8.1)	1,289 (14.8)	6,151 (13.7)	2,751 (12.7)	615 (8.3)	2,785 (17.5)
≤ 20 Years	20,643 (36.3)	9,120 (44.9)	421 (7.0)	8,620 (39.6)	2,462 (28.3)	9,015 (20.1)	2,214 (10.2)	3,151 (42.6)	3,650 (23.0)
21–30 Years	24,414 (42.9)	6,843 (33.7)	3,779 (63.2)	9,592 (44.0)	4,157 (47.8)	25,590 (57.0)	14,325 (66.1)	3,158 (42.7)	8,107 (51.1)
> 30 Years	3,219 (5.7)	847 (4.2)	634 (10.6)	1,123 (5.2)	613 (7.1)	3,200 (7.1)	1,895 (8.8)	231 (3.1)	1,074 (6.8)
Missing	1,814 (3.2)	818 (4.0)	129 (2.2)	690 (3.2)	174 (2.0)	973 (2.2)	474 (2.2)	236 (3.2)	263 (1.7)
Number of children									
0 children (nulliparous)	6,766 (11.9)	2,691 (13.2)	1,020 (17.1)	1,762 (8.1)	1,289 (14.8)	6,151 (13.7)	2,751 (12.7)	615 (8.3)	2,785 (17.5)
1 child	6,547 (11.5)	3,219 (15.8)	711 (11.9)	1,519 (7.0)	1,088 (12.5)	4,930 (11.0)	2,446 (11.3)	506 (6.9)	1,978 (12.5)
2–3 children	21,584 (38.0)	7,197 (35.4)	3,297 (55.1)	6,938 (31.8)	4,124 (47.4)	22,206 (49.4)	12,098 (55.9)	2,626 (35.5)	7,482 (47.1)
≥ 4 children	21,123 (37.2)	6,887 (33.9)	920 (15.4)	11,177 (51.3)	2,110 (24.3)	11,330 (25.2)	4,226 (19.5)	3,578 (48.4)	3,526 (22.2)
Missing	836 (1.5)	325 (1.6)	35 (0.6)	391 (1.9)	84 (1.0)	312 (0.7)	138 (0.6)	66 (0.9)	108 (0.7)
Menopausal status									
Pre-menopausal	5,788 (10.2)	2,143 (10.6)	752 (12.6)	2,108 (9.7)	775 (8.9)	8,137 (18.1)	3,410 (15.7)	1,645 (22.3)	3,082 (19.4)
Natural menopause	27,586 (48.5)	8,089 (39.8)	3,408 (57.0)	11,485 (52.7)	4,563 (52.5)	21,668 (48.2)	11,260 (52.0)	3,067 (41.5)	7,341 (46.2)
Oophorectomy	8,331 (14.7)	3,526 (17.4)	761 (12.7)	2,599 (11.9)	1,440 (16.6)	6,708 (14.9)	3,209 (14.8)	1,106 (15.0)	2,393 (15.1)
Hysterectomy	11,344 (20.0)	5,154 (25.4)	650 (10.9)	4,004 (18.4)	1,526 (17.6)	6,251 (13.9)	2,518 (11.6)	1,189 (16.1)	2,544 (16.0)
Period stopped, reason unknown	3,053 (5.4)	1,169 (5.8)	375 (6.3)	1,171 (5.4)	333 (3.8)	1,861 (4.1)	1,122 (5.2)	295 (4.0)	444 (2.8)
Missing	754 (1.3)	238 (1.2)	37 (0.6)	420 (1.9)	58 (0.7)	304 (0.7)	140 (0.7)	89 (1.2)	75 (0.5)
Menopausal hormone use									
Never estrogen use, with or without past or current progesterone use	30,542 (53.7)	11,643 (57.3)	3,233 (54.0)	11,835 (54.3)	3,796 (43.7)	21,871 (48.7)	10,372 (47.9)	4,413 (59.7)	7,086 (44.6)
Past estrogen use, with or without past progesterone use	9,919 (17.5)	3,914 (19.3)	755 (12.6)	3,666 (16.8)	1,567 (18.0)	6,097 (13.6)	2,683 (12.4)	1,018 (13.8)	2,396 (15.1)
Current estrogen use, with or without past or current progesterone use	12,787 (22.5)	3,466 (17.1)	1,806 (30.2)	4,467 (20.5)	3,030 (34.9)	15,564 (34.6)	7,778 (35.9)	1,692 (22.9)	6,094 (38.4)
Missing	3,608 (6.4)	1,296 (6.4)	189 (3.2)	1,819 (8.4)	302 (3.5)	1,397 (3.1)	826 (3.8)	268 (3.6)	303 (1.9)
Alcohol use in past year									
None	33,922 (59.7)	12,207 (60.1)	4,197 (70.2)	13,597 (62.4)	3,875 (44.6)	27,647 (61.5)	17,055 (78.7)	4,669 (63.2)	5,923 (37.3)
Any alcohol use	20,369 (35.8)	7,161 (35.2)	1,552 (25.9)	7,255 (33.3)	4,378 (50.4)	17,216 (38.3)	4,571 (21.1)	2,711 (36.7)	9,934 (62.6)
Missing	2565 (4.5)	951 (4.7)	234 (3.9)	935 (4.3)	442 (5.1)	66 (0.2)	33 (0.2)	11 (0.2)	22 (0.1)
Ever smoked 20 or more packs									
No	30,596 (53.8)	9,021 (44.4)	3,984 (66.6)	13,568 (62.3)	3,981 (45.8)	24,970 (55.6)	14,820 (68.4)	3,260 (44.1)	6,890 (43.4)
Yes, and quit smoking	16,478 (29.0)	6,883 (33.9)	1,407 (23.5)	5,070 (23.3)	3,103 (35.7)	13,207 (29.4)	4,617 (21.3)	2,341 (31.7)	6,249 (39.4)
Yes, and currently smoking	8,244 (14.5)	4,085 (20.1)	535 (8.9)	2,121 (9.7)	1,489 (17.1)	6,287 (14.0)	1,950 (9.0)	1,708 (23.1)	2,629 (16.6)
Missing	1,538 (2.7)	330 (1.6)	57 (1.0)	1028 (4.7)	122 (1.4)	465 (1.0)	272 (1.3)	82 (1.1)	111 (0.7)
Energy intake in past year (kcal/day), quintile									
Quintile 1: 425.20–1228.11	12,183 (21.4)	5,226 (25.7)	1,255 (21.0)	3,855 (17.7)	1,837 (21.1)	7,646 (17.0)	3,812 (17.6)	981 (13.3)	2,853 (18.0)
Quintile 2: 1228.13–1588.09	10,167 (17.9)	3,640 (17.9)	1,371 (22.9)	3,296 (15.1)	1,846 (21.2)	9,665 (21.5)	4,983 (23.0)	1,050 (14.2)	3,632 (22.9)
Quintile 3: 1588.10–1975.66	9,888 (17.4)	3,324 (16.4)	1,319 (22.1)	3,496 (16.1)	1,738 (20.0)	9,944 (22.1)	5,107 (23.6)	1,201 (16.3)	3,636 (22.9)
Quintile 4: 1975.67–2581.16	10,095 (17.8)	3,311 (16.3)	1,108 (18.5)	4,021 (18.5)	1,643 (18.9)	9,735 (21.7)	4,657 (21.5)	1,629 (22.0)	3,449 (21.7)
Quintile 5: 2581.28–7741.08	11,958 (21.0)	3,867 (19.0)	696 (11.6)	6,184 (28.4)	1,189 (13.7)	7,873 (17.5)	3,067 (14.2)	2,519 (34.1)	2,287 (14.4)
Missing	2,565 (4.5)	951 (4.7)	234 (3.9)	935 (4.3)	442 (5.1)	66 (0.2)	33 (0.2)	11 (0.2)	22 (0.1)
Moderate to vigorous physical activity in past year^a^ (hours/day), quintile									
0 (no activity)	4,573 (8.0)	1,187 (5.8)	149 (2.5)	2,892 (13.3)	343 (3.9)	836 (1.9)	381 (1.8)	176 (2.4)	279 (1.8)
Quartile 1: 0.11–0.36	19,187 (33.8)	7,698 (37.9)	1,952 (32.6)	7,202 (33.1)	2,315 (26.6)	11,900 (26.5)	7,186 (33.2)	1,854 (25.1)	2,860 (18.0)
Quartile 2: 0.46–0.71	12,181 (21.4)	4,635 (22.8)	1,424 (23.8)	4,251 (19.5)	1,854 (21.3)	10,019 (22.3)	5,129 (23.7)	1,686 (22.8)	3,204 (20.2)
Quartile 3: 0.82–1.21	8,144 (14.3)	2,839 (14.0)	1,001 (16.7)	2,673 (12.3)	1,619 (18.6)	8,568 (19.1)	3,848 (17.8)	1,424 (19.3)	3,296 (20.8)
Quartile 4: 1.32–13.29	10,543 (18.5)	3,057 (15.1)	1,368 (22.9)	3,690 (16.9)	2,407 (27.7)	13,112 (29.2)	4,854 (22.4)	2,138 (28.9)	6,120 (38.5)
Missing	2,228 (3.9)	903 (4.4)	89 (1.5)	1,079 (5.0)	157 (1.8)	494 (1.1)	261 (1.2)	113 (1.5)	120 (0.8)
Body mass index category									
Underweight	951 (1.7)	204 (1.0)	381 (6.4)	173 (0.8)	190 (2.2)	1,695 (3.8)	1,129 (5.2)	75 (1.0)	491 (3.1)
Normal	19,144 (33.7)	4,699 (23.1)	4,093 (68.4)	6,465 (29.7)	3,867 (44.5)	24,114 (53.7)	13,420 (62.0)	2,248 (30.4)	8,446 (53.2)
Overweight	19,829 (34.9)	7,215 (35.5)	1,227 (20.5)	8,650 (39.7)	2,707 (31.1)	12,012 (26.7)	5,396 (24.9)	2,365 (32.0)	4,251 (26.8)
Obese	15,923 (28.0)	7,479 (36.8)	271 (4.5)	6,247 (28.7)	1,907 (21.9)	6,701 (14.9)	1,468 (6.8)	2,615 (35.4)	2,618 (16.5)
Missing	1,009 (1.8)	722 (3.6)	11 (0.2)	252 (1.2)	24 (0.3)	407 (0.9)	246 (1.1)	88 (1.2)	73 (0.5)
Weight change from age 21 to baseline (per 5 kg), quintile									
Quintile 1: −26.10–0.72	7,834 (13.8)	1,432 (7.1)	2,213 (37.0)	2,455 (11.3)	1,727 (19.9)	11,287 (25.1)	6,495 (30.0)	927 (12.5)	3,865 (24.3)
Quintile 2: 0.81–1.71	7,552 (13.3)	1,670 (8.2)	1,605 (26.8)	2,777 (12.8)	1,486 (17.1)	9,775 (21.8)	5,663 (26.2)	1,012 (13.7)	3,100 (19.5)
Quintile 3: 1.80–2.79	10,385 (18.3)	3,075 (15.1)	1,168 (19.5)	4,453 (20.4)	1,670 (19.2)	9,476 (21.1)	4,847 (22.4)	1,424 (19.3)	3,205 (20.2)
Quintile 4: 2.88–4.41	11,004 (19.4)	4,160 (20.5)	546 (9.1)	4,778 (21.9)	1,504 (17.3)	6,634 (14.8)	2,650 (12.2)	1,438 (19.5)	2,546 (16.0)
Quintile 5: 4.50–29.61	14,029 (24.7)	6,983 (34.4)	202 (3.4)	4,964 (22.8)	1,868 (21.5)	5,702 (12.7)	977 (4.5)	2,118 (28.7)	2,607 (16.4)
Missing	6,052 (10.6)	2,999 (14.8)	249 (4.2)	2,360 (10.8)	440 (5.1)	2,055 (4.6)	1,027 (4.7)	472 (6.4)	556 (3.5)

All values are frequencies and column percentages unless otherwise noted

^a^ In the study questionnaire, examples of moderate activity included housework, brisk walking, golfing, bowling, bicycling on level ground and gardening. Examples of vigorous activity included moving heavy furniture, loading or unloading trucks, shoveling, weightlifting, or equivalent manual labor

### Distribution of the residential segregation measures

In California, African American and Latina females were more likely to live in less economically (income segregation quintile 1 [Q1]: 48.4% and 21.3%, respectively) and less educationally privileged neighborhoods (education segregation Q1: 26.5% and 36.8% respectively) than Japanese American and White females (income segregation Q1: 8.7% and 8.8%; education segregation Q1: 4.8% and 9.0%, respectively) (Table 3). African American, Japanese American, and Latina females were more likely to live in neighborhoods with high concentrations of low-income Black, Asian American or Pacific Islander, and Hispanic households, respectively (racialized income segregation Q1: 87.2%, 29.0%, 36.1%, respectively), than in neighborhoods with high concentrations of high-income White households. In Hawai‘i, Native Hawaiian females lived in less economically or less educationally privileged neighborhoods than Japanese American or White females (income segregation Q1: 14.5% vs. 10.4% and 13.4%, respectively; education segregation Q1: 18.3% vs. 10.0% and 6.5%, respectively). Japanese American and Native Hawaiian females were more likely to live in neighborhoods with high concentrations of high-income White households (racialized income segregation Q5: 24.0%, 19.5%, respectively) compared to neighborhoods with high concentrations of low-income Japanese American and Native Hawaiian households, respectively.

**Table 3:** Distribution of neighborhood measures by state among all female participants, those diagnosed with breast cancer, and by race and ethnicity in the Multiethnic Cohort, 1993–2019

	California						Hawai‘i				
	Overall	Breast Cancer Case	African American	Japanese American	Latina	White	Overall	Breast Cancer Case	Japanese American	Native Hawaiian	White
	n = 56,856	n = 3,640	n = 20,319	n = 5,983	n = 21,787	n = 8,695	n = 44,929	n = 3,741	n = 21,659	n = 7,391	n = 15,879
Income segregation^a^											
Quintile 1—Least privileged	15,765 (27.7)	911 (25.0)	9,837 (48.4)	519 (8.7)	4,635 (21.3)	764 (8.8)	5,455 (12.1)	427 (11.4)	2,253 (10.4)	1,072 (14.5)	2,130 (13.4)
Quintile 2	13,991 (24.6)	805 (22.1)	4,911 (24.2)	851 (14.2)	6,814 (31.3)	1,405 (16.2)	7,208 (16.0)	580 (15.5)	3,105 (14.3)	1,520 (20.6)	2,583 (16.3)
Quintile 3	10,889 (19.2)	691 (19.0)	2,115 (10.4)	1440 (24.1)	5,448 (25.0)	1,868 (21.5)	8,916 (19.8)	743 (19.9)	3,914 (18.1)	1,534 (20.8)	3,468 (21.8)
Quintile 4	9,406 (16.5)	704 (19.3)	2,306 (11.4)	1,590 (26.6)	3,208 (14.7)	2,277 (26.2)	9,049 (20.1)	777 (20.8)	4,657 (21.5)	1,606 (21.7)	2,786 (17.6)
Quintile 5—Most privileged	6,804 (12.0)	529 (14.5)	1,150 (5.7)	1,583 (26.5)	1,681 (7.7)	2,381 (27.4)	13,660 (30.4)	1,162 (31.1)	7,537 (34.8)	1,552 (21.0)	4,571 (28.8)
Missing	1 (0)	0 (0)	0 (0)	0 (0)	1 (0)	0 (0)	641 (1.4)	52 (1.4)	193 (0.9)	107 (1.5)	341 (2.2)
Education segregation^b^											
Quintile 1—Least privileged	14,472 (25.5)	742 (20.4)	5,374 (26.5)	286 (4.8)	8,019 (36.8)	782 (9.0)	4,545 (10.1)	388 (10.4)	2,159 (10.0)	1,355 (18.3)	1,031 (6.5)
Quintile 2	16,279 (28.6)	1,021 (28.1)	7,467 (36.8)	1,007 (16.8)	6,224 (28.6)	1,563 (18.0)	8,412 (18.7)	641 (17.1)	3,878 (17.9)	1,767 (23.9)	2,767 (17.4)
Quintile 3	11,016 (19.4)	716 (19.7)	3,446 (17.0)	1,554 (26.0)	3,991 (18.3)	2,007 (23.1)	10,062 (22.4)	875 (23.4)	5,266 (24.3)	1,511 (20.4)	3,285 (20.7)
Quintile 4	9,574 (16.8)	727 (20.0)	2,968 (14.6)	1,836 (30.7)	2,412 (11.1)	2,338 (26.9)	9,883 (22.0)	839 (22.4)	4,660 (21.5)	1,452 (19.7)	3,771 (23.8)
Quintile 5—Most privileged	5,515 (9.7)	434 (11.9)	1,064 (5.2)	1,300 (21.7)	1,141 (5.2)	2,005 (23.1)	11,475 (25.5)	956 (25.6)	5,569 (25.7)	1,207 (16.3)	4,699 (29.6)
Missing	0 (0)	0 (0)	0 (0)	0 (0)	0 (0)	0 (0)	552 (1.2)	42 (1.1)	127 (0.6)	99 (1.3)	326 (2.1)
Racialized income segregation											
Quintile 1—Least privileged	NA	NA	17,717 (87.2)	1,734 (29.0)	7,867 (36.1)	NA	NA	NA	2,309 (10.7)	1,217 (16.5)	NA
Quintile 2	NA	NA	969 (4.8)	753 (12.6)	6,033 (27.7)	NA	NA	NA	4,139 (19.1)	1,604 (21.7)	NA
Quintile 3	NA	NA	795 (3.9)	1,259 (21.0)	4,313 (19.8)	NA	NA	NA	5,053 (23.3)	1,448 (19.6)	NA
Quintile 4	NA	NA	543 (2.7)	1,121 (18.7)	2,316 (10.6)	NA	NA	NA	4,772 (22.0)	1,576 (21.3)	NA
Quintile 5—Most privileged	NA	NA	295 (1.5)	1,116 (18.7)	1,257 (5.8)	NA	NA	NA	5,193 (24.0)	1,439 (19.5)	NA
Missing	NA	NA	0 (0)	0 (0)	1 (0)	NA	NA	NA	193 (0.9)	107 (1.5)	NA
Race and ethnicity segregation											
Quintile 1—Least privileged	NA	NA	17,947 (88.3)	2,454 (41.0)	9,962 (45.7)	NA	NA	NA	7,358 (34.0)	2,042 (27.6)	NA
Quintile 2	NA	NA	601 (3.0)	946 (15.8)	5,241 (24.1)	NA	NA	NA	5,614 (25.9)	1,385 (18.7)	NA
Quintile 3	NA	NA	903 (4.4)	1,111 (18.6)	3,958 (18.2)	NA	NA	NA	3,153 (14.6)	1,659 (22.5)	NA
Quintile 4	NA	NA	544 (2.7)	980 (16.4)	1,942 (8.9)	NA	NA	NA	3,896 (18.0)	1,551 (21.0)	NA
Quintile 5—Most privileged	NA	NA	324 (1.6)	492 (8.2)	684 (3.1)	NA	NA	NA	1,511 (7.0)	655 (8.9)	NA
Missing	NA	NA	0 (0)	0 (0)	0 (0)	NA	NA	NA	127 (0.6)	99 (1.3)	NA

All values are frequencies and column percentages unless otherwise noted. Index of Concentration at the Extremes race and ethnicity segregation measures are modeled among race-specific populations, e.g., Non-Hispanic White (most privileged) and Non-Hispanic Black (least privileged) indices are used in race and ethnicity segregation models among African American females. Among the California participants, Native Hawaiian females were excluded from the stratified analyses by race and ethnicity due to small numbers (n = 72). “NA” = Not Applicable; race and ethnicity segregation measures not defined for the overall population or White female participants.

^a^ The quintiles for the income segregation measures were defined using the following state-specific percentile cut points: California—p20, −0.24, p40, −0.11, p60, −0.0004, p80, 0.15; Hawai‘i—p20, −0.15, p40, −0.041, p60, 0.042, p80, 0.19.

^b^The quintiles for the education segregation measures were defined using the following state-specific percentile cut points: California—p20, −0.28, p40, −0.078, p60, 0.081, p80, 0.27; Hawai‘i—p20, −0.19, p40, −0.040, p60, 0.068, p80, 0.23

### Associations with income and education residential segregation measures

Among California female participants, those who lived in more privileged neighborhoods with higher concentrations of high-income households had an increased risk of breast cancer in fully adjusted models (income segregation Q5 vs. Q1: HR 1.12, 95% CI 1.00–1.25; *P*_trend_ = 0.001) (Table 4). Increased risk was highest among Latina females (income segregation Q4 vs. Q1: HR 1.23, 95% CI 1.01–1.50; *P*_trend_ = 0.01). Similarly, there was increased risk among California female participants living in neighborhoods with higher concentrations of higher educated residents (education segregation Q5 vs. Q1: HR 1.15, 95% CI 1.01–1.31; *P*_trend_ = 0.01). Increased risks were highest among Latina females (education segregation Q5 vs. Q1: HR 1.27, 95% CI 0.99–1.62; *P*_trend_ = 0.004) compared to other racial and ethnic groups. Among White females, a statistically significant trend in the age-adjusted model (*P*_trend_ = 0.02) was attenuated when adjusting for breast cancer risk factors (*P*_trend_ = 0.18). In Hawai‘i, there were no statistically significant associations of income or education segregation measures with breast cancer risk overall or by race and ethnicity (Table 5).

**Table 4:** Hazard ratios for invasive breast cancer for associations with income, education, racialized income, and race and ethnicity segregation, overall and by race and ethnicity among California Multiethnic Cohort female participants, (n = 56,856), 1993–2019

	Overall			African American			Japanese American			Latina			White		
	n = 56,856			n = 20,319			n = 5,983			n = 21,787			n = 8,695		
	Breast cancer cases n = 3,640	Age + race/eth HR (95% CI)	Full model HR (95% CI)	Breast cancer cases n = 1,392	Age adjusted HR (95% CI)	Full model HR (95% CI)	Breast cancer cases n = 440	Age adjusted HR (95% CI)	Full model HR (95% CI)	Breast cancer cases n = 1,133	Age adjusted HR (95% CI)	Full model HR (95% CI)	Breast cancer cases n = 663	Age adjusted HR (95% CI)	Full model HR (95% CI)
Income segregation															
Quintile 1—Least privileged	911	1.00	1.00	616	1.00	1.00	26	1.00	1.00	205	1.00	1.00	61	1.00	1.00
Quintile 2	805	1.03 (0.94–1.13)	1.00 (0.91–1.10)	352	1.10 (0.97–1.25)	1.07 (0.94–1.21)	52	1.21 (0.75–1.93)	1.18 (0.72–1.95)	318	1.04 (0.87–1.25)	1.00 (0.84–1.21)	80	**0.69 (0.50**–**0.96)**	**0.69 (0.49**–**0.95)**
Quintile 3	691	**1.12 (1.01**–**1.25)**	1.07 (0.96–1.19)	152	1.11 (0.92–1.34)	1.06 (0.88–1.28)	98	1.25 (0.79–1.97)	1.21 (0.75–1.95)	208	**1.25 (1.03**–**1.50)**	1.16 (0.96–1.40)	131	0.82 (0.61–1.10)	0.82 (0.61–1.11)
Quintile 4	704	**1.24 (1.13–1.37)**	**1.15 (1.04–1.28)**	183	1.16 (0.99–1.36)	1.09 (0.91–1.31)	139	1.54 (0.99–2.37)	1.45 (0.91–2.31)	199	**1.38 (1.13–1.67)**	**1.23 (1.01**–**1.50**)	181	0.93 (0.70–1.24)	0.92 (0.68–1.23)
Quintile 5—Most privileged	529	**1.23 (1.10–1.37)**	**1.12 (1.00–1.25)**	89	1.11 (0.95–1.29)	1.03 (0.88–1.20)	125	1.37 (0.88–2.14)	1.25 (0.78–2.01)	103	**1.32 (1.06–1.66)**	1.16 (0.92–1.46)	210	1.00 (0.75–1.32)	0.95 (0.70–1.28)
P trend		< 0.0001	0.001		0.02	0.28		0.03	0.17		< 0.0001	0.01		0.10	0.26
Education segregation															
Quintile 1—Least privileged	742	1.00	1.00	321	1.00	1.00	15	.	.	354	1.00	1.00	49	1.00	1.00
Quintile 2	1,021	**1.15 (1.05**–**1.26)**	**1.11 (1.01**–**1.22)**	527	**1.15 (1.01**–**1.30)**	1.13 (0.99–1.28)	68	.	.	316	1.14 (0.97–1.35)	1.09 (0.93–1.29)	106	1.04 (0.76–1.44)	1.01 (0.73–1.39)
Quintile 3	716	**1.17 (1.05**–**1.30)**	1.10 (0.99–1.22)	234	1.09 (0.92–1.29)	1.05 (0.88–1.24)	105	.	.	241	**1.36 (1.15**–**1.61)**	**1.26 (1.06**–**1.50)**	134	1.01 (0.74–1.37)	0.94 (0.69–1.29)
Quintile 4	727	**1.28 (1.16**–**1.42)**	**1.18 (1.06**–**1.30)**	241	**1.23 (1.06**–**1.44)**	1.15 (0.97–1.36)	135	.	.	147	**1.35 (1.12**–**1.63)**	1.21 (0.99–1.47)	202	1.29 (0.96–1.73)	1.19 (0.88–1.62)
Quintile 5—Most privileged	434	**1.29 (1.14**–**1.47)**	**1.15 (1.01**–**1.31)**	69	1.01 (0.83–1.22)	0.91 (0.74–1.13)	117	.	.	75	**1.43 (1.12**–**1.82)**	1.27 (0.99–1.62)	172	1.22 (0.90–1.66)	1.08 (0.79–1.50)
P trend		< 0.0001	0.01		0.17	0.95					< 0.0001	0.004		0.02	0.18
Racialized income segregation															
Quintile 1—Least privileged	NA	NA	NA	1,222	1.00	1.00	106	1.00	1.00	339	1.00	1.00	NA	NA	NA
Quintile 2	NA	NA	NA	57	0.87 (0.69–1.09)	0.84 (0.68–1.05)	52	1.15 (0.85–1.56)	1.17 (0.86–1.59)	310	**1.19 (1.00**–**1.41)**	1.15 (0.97–1.37)	NA	NA	NA
Quintile 3	NA	NA	NA	60	1.14 (0.89–1.46)	1.16 (0.90–1.48)	96	1.17 (0.87–1.58)	1.15 (0.85–1.55)	252	**1.34 (1.14**–**1.58)**	**1.26 (1.07**–**1.49)**	NA	NA	NA
Quintile 4	NA	NA	NA	33	0.95 (0.67–1.35)	0.92 (0.65–1.31)	94	1.28 (0.98–1.67)	1.19 (0.91–1.57)	159	**1.56 (1.30**–**1.88)**	**1.41 (1.16**–**1.70)**	NA	NA	NA
Quintile 5—Most privileged	NA	NA	NA	20	0.99 (0.60–1.62)	0.95 (0.59–1.55)	92	1.25 (0.93–1.68)	1.13 (0.83–1.53)	73	**1.30 (1.02**–**1.65)**	1.16 (0.91–1.48)	NA	NA	NA
P trend	NA	NA	NA		0.98	0.68		0.04	0.23		< 0.0001	0.004	NA	NA	NA
Race and ethnicity segregation															
Quintile 1—Least privileged	NA	NA	NA	1,244	1.00	1.00	151	1.00	1.00	451	1.00	1.00	NA	NA	NA
Quintile 2	NA	NA	NA	39	1.05 (0.78–1.41)	1.07 (0.80–1.44)	76	1.34 (0.98–1.83)	1.29 (0.95–1.77)	278	1.16 (0.98–1.36)	1.11 (0.94–1.31)	NA	NA	NA
Quintile 3	NA	NA	NA	54	0.99 (0.75–1.30)	1.01 (0.77–1.33)	86	1.27 (0.99–1.62)	1.21 (0.94–1.56)	232	**1.28 (1.09**–**1.50)**	**1.20 (1.02**–**1.40)**	NA	NA	NA
Quintile 4	NA	NA	NA	33	0.96 (0.64–1.44)	0.94 (0.62–1.41)	72	1.16 (0.87–1.53)	1.08 (0.81–1.45)	128	**1.42** **(1.17**–**1.73)**	**1.30 (1.07**–**1.58)**	NA	NA	NA
Quintile 5—Most privileged	NA	NA	NA	22	1.01 (0.66–1.54)	0.99 (0.65–1.51)	55	**1.76 (1.31**–**2.38)**	**1.55 (1.14**–**2.12)**	44	**1.40** **(1.02**–**1.90)**	1.25 (0.92–1.71)	NA	NA	NA
P trend	NA	NA	NA		0.86	0.87		0.01	0.06		<.0001	0.004	NA	NA	NA

Full model adjusting for age at baseline, education, family history of breast cancer, age at first birth, age at menarche, number of children, menopausal status, menopausal hormone use, alcohol use, smoking status, energy intake, physical activity, body mass index, and weight change. Full models among the overall population additionally include race and ethnicity as a strata variable. All models account for clustering at the census tract level. Index of Concentration at the Extremes race and ethnicity segregation measures are modeled among race-specific populations, e.g., Non-Hispanic White (most privileged) and Non-Hispanic Black (least privileged) indices are used in race and ethnicity segregation models among African American females. Among the California participants, Native Hawaiian females were excluded from the stratified analyses by race and ethnicity due to small numbers (*n* = 72). ".." = Not reportable due to insufficient case count among referent group (n < 20). “NA” = Not Applicable; race and ethnicity segregation measures not defined for overall population or White females. **Bold** values are statistically significant at *P* < 0.05

Abbreviations: CI, confidence interval; Eth, ethnicity; HR, hazard ratio

**Table 5:** Hazard ratios for invasive breast cancer for associations with income, education, racialized income, and race and ethnicity segregation, overall and by race and ethnicity among Hawai‘i Multiethnic Cohort female participants, (n = 44,929), 1993–2019

	Overall			Japanese American			Native Hawaiian			White		
	n = 44,929			n = 21,659			n = 7,391			n = 15,879		
	Breast cancer cases *n* = 3741	Age + race/eth HR (95% CI)	Full model HR (95% CI)	Breast cancer cases *n* = 1803	Age adjusted HR (95% CI)	Full model HR (95% CI)	Breast cancer cases *n* = 775	Age adjusted HR (95% CI)	Full model HR (95% CI)	Breast cancer cases *n* = 1631	Age adjusted HR (95% CI)	Full model HR (95% CI)
Income Segregation												
Quintile 1—Least privileged	427	1.00	1.00	162	1.00	1.00	106	1.00	1.00	159	1.00	1.00
Quintile 2	580	1.02 (0.90–1.16)	1.01 (0.89–1.14)	259	1.15 (0.94–1.41)	1.16 (0.95–1.41)	153	1.06 (0.85–1.32)	1.05 (0.85–1.30)	168	0.85 (0.67–1.08)	0.83 (0.66–1.04)
Quintile 3	743	1.05 (0.93–1.19)	1.05 (0.93–1.18)	326	1.15 (0.93–1.41)	1.14 (0.93–1.41)	161	1.07 (0.85–1.33)	1.09 (0.87–1.36)	256	0.95 (0.80–1.13)	0.93 (0.79–1.09)
Quintile 4	777	1.06 (0.94–1.20)	1.05 (0.93–1.18)	391	1.13 (0.93–1.39)	1.12 (0.91–1.38)	178	1.12 (0.91–1.38)	1.12 (0.90–1.38)	208	0.96 (0.81–1.14)	0.93 (0.79–1.10)
Quintile 5—Most privileged	1,162	1.05 (0.94–1.17)	1.01 (0.90–1.13)	649	1.12 (0.93–1.34)	1.08 (0.90–1.30)	168	1.06 (0.85–1.33)	1.07 (0.85–1.35)	345	0.96 (0.80–1.15)	0.89 (0.75–1.05)
P trend		0.26	0.79		0.44	0.91		0.58	0.48		0.66	0.75
Education segregation												
Quintile 1—Least privileged	388	1.00	1.00	167	1.00	1.00	150	1.00	1.00	71	1.00	1.00
Quintile 2	641	0.93 (0.83–1.04)	0.92 (0.82–1.03)	271	0.93 (0.75–1.14)	0.90 (0.73–1.11)	176	0.90 (0.75–1.08)	0.91 (0.77–1.09)	194	1.00 (0.82–1.23)	0.99 (0.81–1.22)
Quintile 3	875	1.06 (0.94–1.19)	1.05 (0.94–1.17)	472	1.14 (0.94–1.39)	1.11 (0.91–1.36)	169	1.01 (0.83–1.23)	1.03 (0.85–1.24)	234	1.01 (0.82–1.25)	0.98 (0.80–1.21)
Quintile 4	839	1.03 (0.91–1.16)	1.00 (0.89–1.12)	408	1.10 (0.89–1.35)	1.04 (0.85–1.28)	146	0.87 (0.72–1.05)	0.90 (0.75–1.08)	285	1.07 (0.86–1.35)	1.04 (0.83–1.30)
Quintile 5—Most privileged	956	1.02 (0.92–1.14)	0.97 (0.87–1.08)	477	1.07 (0.88–1.30)	1.00 (0.82–1.22)	126	0.91 (0.75–1.12)	0.94 (0.77–1.16)	353	1.05 (0.86–1.28)	0.97 (0.78–1.20)
P trend		0.24	0.95		0.22	0.71		0.40	0.56		0.42	0.80
Racialized income segregation												
Quintile 1—least privileged	NA	NA	NA	169	1.00	1.00	121	1.00	1.00	NA	NA	NA
Quintile 2	NA	NA	NA	318	1.04 (0.85–1.27)	1.02 (0.84–1.25)	170	1.07 (0.86–1.33)	1.10 (0.89–1.35)	NA	NA	NA
Quintile 3	NA	NA	NA	424	1.12 (0.93–1.35)	1.11 (0.92–1.33)	178	1.23 (0.98–1.54)	**1.27** **(1.02**–**1.57)**	NA	NA	NA
Quintile 4	NA	NA	NA	445	**1.22** **(1.01**–**1.47)**	1.20 (0.99–1.44)	159	0.98 (0.79–1.22)	1.02 (0.83–1.26)	NA	NA	NA
Quintile 5—Most privileged	NA	NA	NA	431	1.05 (0.88–1.26)	1.02 (0.85–1.22)	138	0.92 (0.72–1.17)	0.96 (0.76–1.21)	NA	NA	NA
P trend	NA	NA	NA		0.17	0.32		0.29	0.49	NA	NA	NA
Race and ethnicity segregation												
Quintile 1—least privileged	NA	NA	NA	637	1.00	1.00	218	1.00	1.00	NA	NA	NA
Quintile 2	NA	NA	NA	439	0.89 (0.78–1.01)	0.89 (0.79–1.02)	166	1.09 (0.90–1.33)	1.11 (0.91–1.34)	NA	NA	NA
Quintile 3	NA	NA	NA	256	0.95 (0.81–1.13)	0.98 (0.82–1.16)	165	0.92 (0.78–1.09)	0.93 (0.79–1.10)	NA	NA	NA
Quintile 4	NA	NA	NA	330	0.99 (0.87–1.13)	1.03 (0.90–1.18)	159	0.92 (0.79–1.07)	0.95 (0.81–1.12)	NA	NA	NA
Quintile 5—most privileged	NA	NA	NA	133	1.03 (0.89–1.19)	1.05 (0.91–1.22)	59	0.82 (0.64–1.04)	0.83 (0.65–1.06)	NA	NA	NA
P trend	NA	NA	NA		0.60	0.28		0.04	0.10	NA	NA	NA

Full model adjusting for age at baseline, education, family history of breast cancer, age at first birth, age at menarche, number of children, menopausal status, menopausal hormone use, alcohol use, smoking status, energy intake, physical activity, body mass index, and weight change. Full models among the overall population additionally include race and ethnicity as a strata variable. All models account for clustering at the census tract level. Index of Concentration at the Extremes race and ethnicity segregation measures are modeled among race-specific populations, e.g., Non-Hispanic White (most privileged) and Non-Hispanic Japanese American (least privileged) indices are used in race and ethnicity segregation models among Japanese American females. “NA” = Not Applicable; race and ethnicity segregation measures not defined for overall population or White females. **Bold** values are statistically significant at *P* < 0.05. Abbreviations: CI, confidence interval, Eth, ethnicity; HR, hazard ratio

### Associations with race-specific segregation measures

In fully adjusted models, California Latina females living in more privileged neighborhoods with higher concentrations of high-incomeWhite households had increased risks of breast cancer compared to those living in neighborhoods with higher concentrations of low-income Hispanic households (racialized income segregation Q4 vs. Q1: HR 1.41, 95% CI 1.16–1.70; *P*_trend_ = 0.004) (Table 4). Latina females living in neighborhoods with higher concentrations of Non-Hispanic White residents also had higher risks of breast cancer than those living in neighborhoods with higher concentrations of Hispanic residents (race and ethnicity segregation Q4 vs. Q1: HR 1.30, 95% CI 1.07–1.58; *P*_trend_ = 0.004). Japanese American females living in more privileged neighborhoods with the highest concentrations of Non-Hispanic White residents than Non-Hispanic Asian American or Pacific Islander persons experienced higher risk of breast cancer (race and ethnicity segregation Q5 vs. Q1: HR 1.55, 95% CI 1.14–2.12) with a borderline statistically significant trend (*P*_trend_ = 0.06). For Native Hawaiian females in Hawai‘i, an increased risk of breast cancer was observed in one category of the racialized income measure (Q3 vs. Q1: HR 1.27, 95% CI 1.02–1.57) (Table 5), but a significant trend was not observed (*P*_trend_ = 0.49).

### Associations by hormone receptor status and stage

For California MEC female participants, there were no statistically significant differences in associations of income segregation or education segregation by hormone receptor status (positive vs. negative) or stage of disease (localized vs. regional & distant) (P_heterogeneity_ > 0.50 for all) (Supplementary Table S1). For Hawai‘i MEC females, there were differences in income segregation associations by stage (P_heterogeneity_ = 0.04) (Supplementary Table S2). Hawai‘i females living in neighborhoods with higher income had higher risk of localized disease (income segregation Q4 vs. Q1: HR 1.17, 95% CI 1.01–1.35; *P*_trend_ = 0.15) and lower risk of regional & distant disease (income segregation Q5 vs. Q1: HR 0.80, 95% CI 0.65–0.99; *P*_trend_ = 0.10).

## Discussion

Overall, we found an increased risk of invasive breast cancer among California MEC female participants living in neighborhoods with high versus low concentrations of privilege as measured by income and education residential segregation. Results were not uniform across race and ethnicity. As prior studies have reported increased risk of breast cancer associated with higher area-level socioeconomic status [6, 33], our study adds to the evidence of the influence of the neighborhood environment by identifying associations with neighborhood residential segregation using the Index of Concentration at the Extremes income, education, racialized income, and race and ethnicity measures.

Prior studies of residential segregation and breast cancer have focused primarily on tumor characteristics, treatment, and mortality [6, 7, 9, 11, 34]. Only the Massachusetts study, examined associations between residential segregation and breast cancer incidence [15] and found a 16% lower breast cancer incidence associated with living in less privileged neighborhoods, measured by income segregation [15]. These results were similar for non-Latina White, non-Latina Black, and Latina females but results were not reported for Asian American and Pacific Islander females due to small sample size. The large, diverse, prospective design of the MEC has allowed us to create race and ethnicity-specific race and racialized income segregation measures for five racial and ethnic groups and to account for many individual-level breast cancer risk factors. Our results suggest similar increasing breast cancer risk with increasing income segregation among California MEC females, overall.

Our results by race and ethnicity showed an increased risk of breast cancer with increasing privilege for each of the four residential segregation measures among Latina females in California, in contrast to the findings in the Massachusetts study of statistically nonsignificant positive associations of the income, race and ethnicity, and racialized income residential segregation measures. Acculturation may explain some of the increased breast cancer risk among Latina women living in privileged neighborhoods. Latina females living in neighborhoods with higher privilege may have greater access to healthcare and timely breast cancer screening which may lead to earlier detection of disease [35]. Also, prior studies have found increased alcohol consumption, a known breast cancer risk factor [36], with increasing acculturation among Latina females [37, 38]. The null association among African American females in our study may be related to that > 85% of African American females in the California MEC reside in neighborhoods in the lowest quintile for race and ethnicity or racialized income segregation measures. Such neighborhoods are characterized as having the lowest concentration of privileged neighborhoods. The sampling framework for the MEC specifically recruited African American adults in Los Angeles County census tracts with high proportions of African American residents [28]. Among White females in California, these findings suggest after adjusting for breast cancer risk factors, income and education segregation do not confer additional effects on breast cancer incidence for this subgroup of the population.

Strengths of our study include the prospective design among a large, racially and ethnically diverse cohort with extensive questionnaire information. The similar findings in our minimally and fully adjusted models indicate the independent effects of the contextual environment with persistence of associations after accounting for sociodemographic, behavioral, and reproductive risk factors. The diversity of the cohort allowed for stratification by distinct Asian American, Native Hawaiian, and Pacific Islander groups. The development of novel Native Hawaiian-specific residential segregation measures using the Index of Concentration at the Extremes adds new insights for the role of neighborhood segregation in breast cancer risk across multiple racial and ethnic populations. This is important as breast cancer incidence varies widely across disaggregated Asian American, Native Hawaiian, and Pacific Islander groups [39, 40]. Other measures of residential segregation, such as the Dissimilarity Index and the Gini Index, allow evaluation of residential segregation by income or race and ethnicity, whereas the Index of Concentration at the Extremes measures allowed for evaluation of residential segregation using a joint income and race and ethnicity measure [41]. There are some limitations as well. While the neighborhood residential segregation measures may have changed during the follow-up period, we focused on baseline measures to capture exposures pertinent to the estimated 8–15-year latency period of breast cancer development [42–44]. We combined regional and distant cancers to represent advanced disease, aiming to increase the statistical power for detecting differences compared to localized disease. We recognize this potentially masks differences in the associations between residential segregation and breast cancer risk for regional and distant cancer. We did not evaluate time-varying measures of residential segregation across the study period. Education information was used to capture individual-level socioeconomic status; information on individual income was not collected. Last, our findings are subject to residual confounding and may not be generalizable to populations outside of California or Hawai‘i.

## Conclusions

This study found that increasing privilege, as measured by income and education residential segregation, is associated with increasing breast cancer risk overall, although variation exists in associations across racial and ethnic groups and states. The pathways by which these segregated neighborhoods influence breast cancer risk warrant future investigations of the contributions of the social and built environment and individual health behaviors and lifestyle factors. These findings extend the limited literature on residential segregation and breast cancer incidence, highlighting the importance of evaluating the role of structural drivers of health and breast cancer.

## Supplementary Information

Below is the link to the electronic supplementary material.

Supplementary file1 (PDF 144 KB)

## Data Availability

The Multiethnic Cohort investigators and institutions affirm their intention to share the research data consistent with all relevant NIH resource/data sharing policies. Data requests should be submitted through the Multiethnic Cohort online data request system at https://www.uhcancercenter.org/for-researchers/mec-data-sharing.

## Abbreviations

AAIR: Age adjusted incidence rate
*A*_*i*_: The number of people or households classified as most privileged in area *i*
BC: Breast cancer
CI: Confidence interval
ER: Estrogen receptor
HR: Hazard ratio
*ICE*_*i*_: Calculated Index of Concentration at the Extremes residential segregation measure in area *i*
IRR: Incidence rate ratio
MEC: Multiethnic Cohort Study
*P*_*i*_: The number of people or households classified as least privileged in area *i*
PR: Progesterone receptor
Q: Quintile
SEER: Surveillance, Epidemiology, and End Results Program
*T*_*i*_: The total population or households in area *i* with household data
