# Supplementary material for: Associations of neighborhood racial and ethnic, economical, and educational segregation with breast cancer risk: The Multiethnic ohort Study
Source: Cancer Causes Control. 2026 Jul 24;37(9):133. doi: 10.1007/s10552-026-02187-4 (PMC13400465; doi:10.1007/s10552-026-02187-4)
Supplement: Supplementary file 1 — Supplementary file1 (PDF 144 KB) [file 10552_2026_2187_MOESM1_ESM.pdf]

Supplementary Table 1. Hazard ratios for the association of income and education segregation measures with invasive breast cancer by hormone receptor status (ER+/PR+, ER-PR-), and stage at diagnosis (localized, advanced) among California Multiethnic Cohort female participants, (n=56,856), 1993-2019.

	Hormone Receptor Status					Stage at Diagnosis				
	Hormone Receptor Positive		Hormone Receptor Negative		P Het[a]	Localized		Regional & Distant		P Het[a]
	n=2,394	HR (95% CI)	n=560	HR (95% CI)		n=2,390	HR (95% CI)	n=1,181	HR (95% CI)	
Income Segregation										
Quintile 1 - Least privileged	553	1.00	154	1.00	0.64	562	1.00	319	1.00	0.53
Quintile 2	529	1.06 (0.93-1.20)	121	0.95 (0.75-1.19)		517	1.01 (0.90-1.14)	275	1.02 (0.87-1.19)	
Quintile 3	440	1.06 (0.93-1.21)	110	1.18 (0.89-1.57)		475	1.13 (0.99-1.28)	207	1.01 (0.84-1.22)	
Quintile 4	492	**1.23 (1.09-1.40)**	99	1.16 (0.88-1.53)		484	**1.20 (1.05-1.38)**	209	1.10 (0.92-1.32)	
Quintile 5 - Most privileged	380	**1.20 (1.04-1.38)**	76	1.23 (0.89-1.69)		352	1.11 (0.96-1.29)	171	1.21 (0.99-1.47)	
P trend		0.0003		0.04			0.01		0.08	
Education Segregation										
Quintile 1 - Least privileged	435	1.00	114	1.00	0.84	447	1.00	269	1.00	0.63
Quintile 2	648	**1.19 (1.04-1.35)**	175	**1.29 (1.02-1.62)**		665	**1.19 (1.05-1.34)**	337	1.04 (0.89-1.22)	
Quintile 3	494	**1.24 (1.08-1.42)**	107	1.21 (0.92-1.60)		486	**1.19 (1.04-1.36)**	221	1.02 (0.85-1.22)	
Quintile 4	509	**1.32 (1.15-1.51)**	102	1.26 (0.95-1.68)		497	**1.26 (1.09-1.45)**	222	1.10 (0.91-1.32)	
Quintile 5 - Most privileged	308	**1.26 (1.08-1.48)**	62	1.42 (0.98-2.04)		295	**1.20 (1.02-1.41)**	132	1.12 (0.90-1.40)	
P trend		0.0004		0.13			0.01		0.20	

All models are including race and ethnicity as a strata variable and adjusting for age at baseline, education, family history of breast cancer, age at first birth, age at menarche, number of children, menopausal status, menopausal hormone use, alcohol use, smoking status, energy intake, physical activity, body mass index, and weight change. All models account for clustering at the census tract level.

[a] Heterogeneity of associations by hormone receptor status and stage were assessed by a competing risk analysis using the Lunn-McNeil approach.

Bold values are statistically significant at $P<0.05$.

Abbreviations: CI, confidence interval; ER, estrogen receptor; HR, hazard ratio; P Het, p-value for heterogeneity; PR, progesterone receptor.

Supplementary Table 2. Hazard ratios for the association of income and education segregation measures with invasive breast cancer by hormone receptor status (ER+/PR+, ER-PR-), and stage at diagnosis (localized, advanced) among Hawai'i Multiethnic Cohort female participants, (n=44,929), 1993-2019.

	Hormone Receptor Status					Stage at Diagnosis				
	Hormone Receptor Positive		Hormone Receptor Negative		P Het[a]	Localized		Regional & Distant		P Het[a]
	n=2,910	HR (95% CI)	n=457	HR (95% CI)		n=2,781	HR (95% CI)	n=908	HR (95% CI)	
Income Segregation										
Quintile 1 - Least privileged	323	1.00	52	1.00	0.78	287	1.00	132	1.00	0.04
Quintile 2	437	1.00 (0.87-1.16)	75	1.08 (0.73-1.59)		422	1.09 (0.95-1.26)	146	0.82 (0.65-1.05)	
Quintile 3	577	1.08 (0.94-1.23)	88	1.01 (0.67-1.52)		558	**1.16 (1.01-1.35)**	177	**0.82 (0.67-0.99)**	
Quintile 4	610	1.09 (0.95-1.25)	97	1.08 (0.76-1.55)		590	**1.17 (1.01-1.35)**	178	0.80 (0.63-1.01)	
Quintile 5 - Most privileged	927	1.06 (0.94-1.20)	132	0.94 (0.64-1.37)		881	1.11 (0.96-1.28)	267	**0.80 (0.65-0.99)**	
P trend		0.18		0.61			0.15		0.10	
Education Segregation										
Quintile 1 - Least privileged	308	1.00	37	1.00	0.28	262	1.00	121	1.00	0.15
Quintile 2	501	0.91 (0.80-1.03)	70	1.04 (0.71-1.54)		468	0.99 (0.87-1.13)	163	**0.77 (0.60-0.99)**	
Quintile 3	668	1.00 (0.89-1.13)	119	**1.47 (1.03-2.11)**		668	**1.16 (1.01-1.33)**	197	0.81 (0.64-1.02)	
Quintile 4	652	0.98 (0.86-1.11)	103	1.28 (0.88-1.84)		620	1.08 (0.94-1.24)	208	0.84 (0.66-1.07)	
Quintile 5 - Most privileged	753	0.97 (0.85-1.09)	117	1.23 (0.85-1.78)		729	1.07 (0.93-1.23)	212	**0.76 (0.59-0.96)**	
P trend		0.90		0.32			0.23		0.08	

All models are including race and ethnicity as a strata variable and adjusting for age at baseline, education, family history of breast cancer, age at first birth, age at menarche, number of children, menopausal status, menopausal hormone use, alcohol use, smoking status, energy intake, physical activity, body mass index, and weight change. All models account for clustering at the census tract level.

[a] Heterogeneity of associations by hormone receptor status and stage were assessed by a competing risk analysis using the Lunn-McNeil approach.

Bold values are statistically significant at $P<0.05$.

Abbreviations: CI, confidence interval; ER, estrogen receptor; HR, hazard ratio; P Het, p-value for heterogeneity; PR, progesterone receptor.
